# Characteristics, Needs, and Perspectives of Individuals Living Alone With Dementia: An Integrative Review

**DOI:** 10.1002/hsr2.70348

**Published:** 2025-01-23

**Authors:** Sara J. Crance, Fang Yu

**Affiliations:** ^1^ Edson College of Nursing and Health Innovation Arizona State University Phoenix Arizona USA; ^2^ Hospice of the Valley Phoenix Arizona USA

**Keywords:** Alzheimer's disease and related dementia, community‐based services, solitary living

## Abstract

**Background:**

The number of individuals living alone with dementia is increasing throughout the world, and they have unique needs that are poorly understood. The aim of this integrative review was to understand the characteristics, needs, and perspectives of individuals living alone with dementia as well as the available community resources to guide future research and clinical practice.

**Methods:**

Electronic (PubMed, CINAHL, and PsycINFO) and manual searches were utilized to identify articles using MeSH terms. Among 5693 identified articles, 31 articles met the eligibility criteria. The quality of the articles was determined utilizing the Joanna Briggs Institute (JBI) Critical Appraisal Tools applicable to the study design.

**Results:**

Individuals living alone with dementia are more likely to be widows, of older age, and lower income, have a higher risk of severe loneliness despite more social contact, higher functional ability, and higher unmet needs despite the use of services, compared to those living with others. Perspectives of individuals living alone with dementia focus on the uncertainty of diagnosis, cognitive fluctuations, and maintaining independence. Lack of specialized services available after diagnosis and barriers to accessing services may lead to decreased independence and increased uncertainty. Overall, there is a lack of specialized services, person‐centered care, and support to meet their unique needs.

**Conclusion:**

Individuals living alone with dementia have unique characteristics, unmet needs, and use of available services, which should be assessed regularly. Future research is needed to develop community‐based, person‐centered services for them and remove barriers to service use.

## Summary

- Clinical implication is to assess the needs and perspectives of individuals living alone with dementia to guide care.
- Research is needed to develop and test person‐centered services to meet the needs and preferences of individuals living alone with dementia.

## 1 Introduction

More than 6.7 million individuals are living with Alzheimer's disease (AD) and AD‐related dementia in the United States in 2023 [1]. Globally, it is estimated that by the year 2050 there will be over 152 million individuals living with dementia [2]. Approximately 65% of individuals with dementia live in the community, with 26% of these individuals living at home alone [3]. As the aging population and the prevalence of dementia increases, the number of individuals living alone with dementia will increase as well [4]. Dementia impairs cognition, functional independence, emotions, personality and behaviors [5, 6], making living alone potentially challenging, and causing safety concerns [5]. Understanding the characteristics, needs, and perspectives of individuals living alone with dementia is crucial to developing supportive care programs, healthcare programs, and accessible resources within their communities. Currently, it has been reported that individuals with dementia who live alone at home represent a unique segment of the population with specific characteristics, needs, and perspectives [7, 8, 9, 10].

The purpose of this integrative literature review was to understand the characteristics, needs, and perspectives of individuals living alone with dementia as well as the available community resources to guide future research and clinical practice. This review fills an important knowledge gap because none of the three similar existing reviews [8, 11, 12] examine needs, services, and preferences focused specifically on individuals living alone with dementia. Two of the reviews are focused on individuals living with cognitive impairment who do not yet have a diagnosis of dementia [8, 11], while the other review focuses only on the needs of individuals living at home and not individuals living alone with dementia [12].

## 2 Methods

To fully study the research questions, both quantitative and qualitative studies were searched using the PRISMA 2020 guidelines and checklist to conduct the search and review. The search engines utilized for this review were PubMed, CINAHL, and PsycINFO, using a combination of MeSH terms and major subject searches. The terms used in the search included combinations of the following words: *dementia (MeSH), Alzheimer's Disease (MeSH), living alone, solitary living*, and *community dwelling* (see Table S1). The search spans the whole time period of each search engine to include foundation articles in the research area. The final search was conducted on October 1, 2024. The quality of the articles was assessed utilizing the Joanna Briggs Institute (JBI) Critical Appraisal Tools [13, 14, 15], which are designed to evaluate the quality of the methods, approach, ethical considerations, and reporting of the studies applicable to the study design (see Table S2).

The inclusion criteria were (1) a study sample of individuals living alone in the community with dementia, (2) a focus on the needs, demographics, community programs and resources, and perspectives of individuals living alone with dementia, and (3) peer‐reviewed original research articles. The exclusion criteria were individuals living in assisted living, long‐term care facilities, or hospice care, health provider perspective, response articles, magazine articles, and articles that were not in English (see Table S4).

## 3 Results

**Figure 1:**
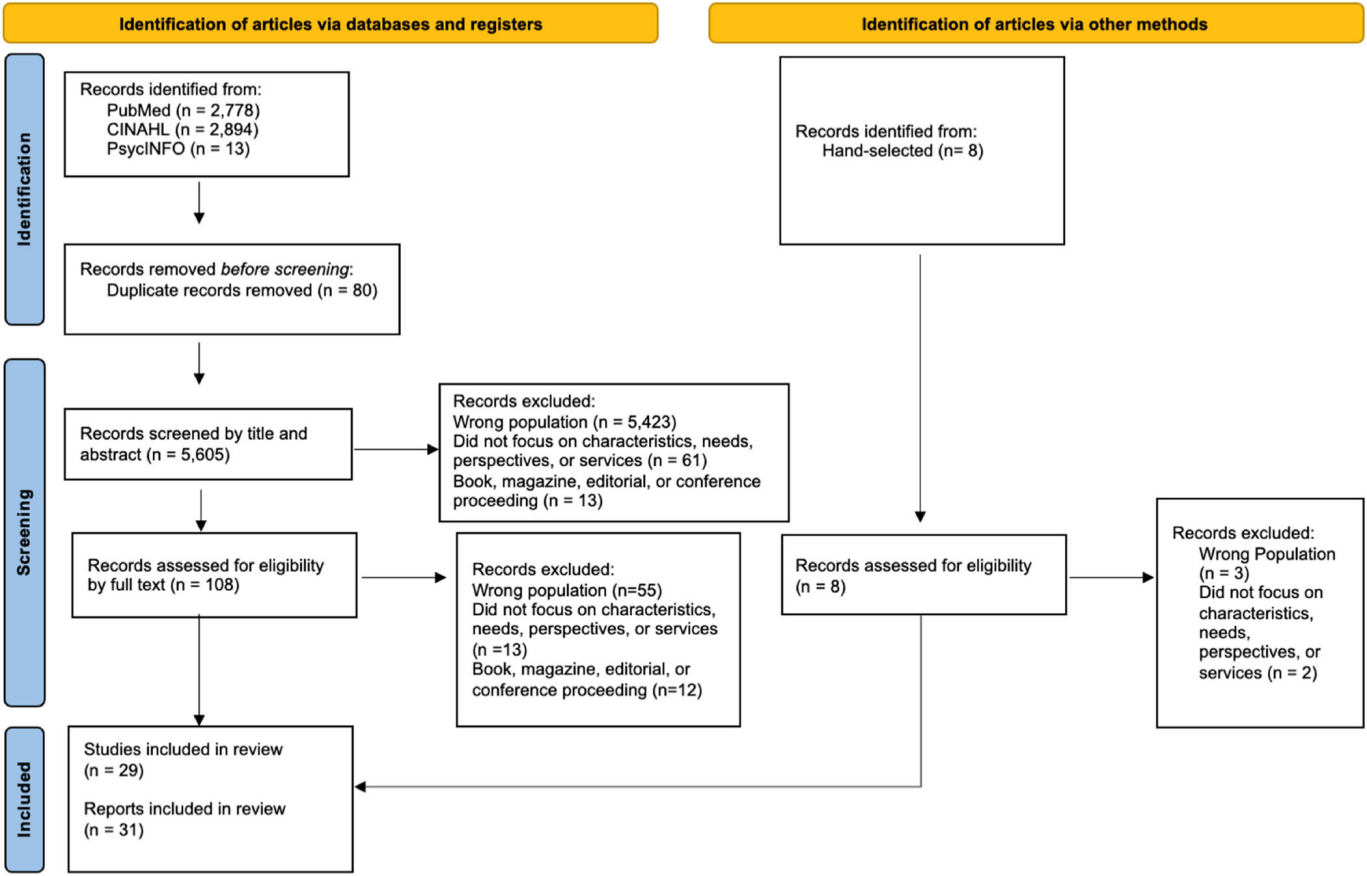
PRISMA flow diagram for article selection.

The initial search produced 5685 articles, and a manual search resulted in 8 articles for a total of 5693, of which 80 were duplicates. Consequently, 5613 articles were screened by title and abstract by the first author (SC), of which 5497 were excluded based on eligibility criteria (see Figure 1). The remaining 116 articles were screened by reading the full text by the first author (S. C.), and 31 articles met the eligibility criteria. As a result, 31 articles were included in the review from 29 studies (see Figure 1).

The 31 articles utilized for this review took place in the United States, *n* = 7 [7, 10, 16, 17, 18, 19, 20], Canada, *n* = 2 [21, 22], France, *n* = 2 [23, 24], Sweden, *n* = 3 [25, 26, 27], Germany, *n* = 2 [9, 28], Australia, *n* = 2 [29, 30], England, Scotland, and Sweden, *n* = 2 [31, 32], Republic of Ireland, *n* = 1 [33], and United Kingdom, *n* = 10 [4, 34, 35, 36, 37, 38, 39, 40, 41, 42] (see Table S2). The articles were a diverse collection of studies: cross‐sectional, *n* = 10 [4, 9, 10, 16, 17, 21, 23, 33, 34, 36], cohort, *n* = 8 [18, 22, 24, 25, 31, 35, 38, 42], and qualitative, *n* = 13 [7, 19, 20, 26, 27, 28, 29, 30, 32, 37, 39, 40, 41], (see Table S2). Three papers from the same study were included in this review [4, 34, 42]. Two of the articles were written by the same author and took place in the same country but included different participants [39, 40]. Additionally, two of the articles were written by the same author and utilized different participants for each of the studies, but they were recruited from the same 5‐year project [27, 32].

Of the 31 articles, 10 articles focused predominantly on the characteristics of individuals living alone with dementia [4, 10, 16, 17, 21, 23, 25, 34, 35, 42]. Additionally, 11 of the articles focused on the needs of individuals living alone with dementia for services, diagnosis, medical management, care, risks, unmet needs, and living arrangements [9, 18, 22, 24, 31, 33, 36, 37, 38, 39, 40]. The remaining 10 articles focused on the perspectives of individuals living alone with dementia regarding diagnosis, social networks, relationships, support, and their environment at home and in their neighborhood [7, 19, 20, 26, 27, 28, 29, 30, 32, 41]. Among the 31 articles, 5 also focused on services available and/or utilized by individuals living with dementia [9, 17, 24, 32, 36] (see Table S2).

### 3.1 Characteristics of Individuals Living Alone With Dementia

Individuals living alone with dementia were more likely to be female, widowed, have a lower income, and were diagnosed with dementia at an older age than those who were living with someone [4, 9, 10, 17, 21, 22, 23, 24, 25, 31, 33, 35, 36, 42]. Only four of the articles included race or ethnicity data [7, 17, 18, 20], with the majority of the participants in all four of the studies identifying as White [7, 17, 18, 20]. Additionally, four of the articles [4, 10, 36, 42] identified a limitation of the study was that the majority of participants were White but did not include race or ethnicity data (two of the articles utilized the same sample) [4, 42]. Individuals living alone with dementia had more social contact and a higher functional ability [4, 10, 16, 42]. Additionally, individuals living alone with dementia were at higher risk of and were more likely to experience severe loneliness and depression [34].

There is variation in the reporting of differences in cognitive ability in individuals living alone with dementia. Mini‐Mental Status Exam scores were reported as not significantly different [21] and lower [23] compared to individuals living with others in two of the studies. However, this variation may be attributable to educational levels because education has an inverse relationship with cognition [43], or statistical significance could be due to the sample size. Other studies determined that individuals living alone with dementia had higher cognitive ability [4, 10, 16, 17, 42]. There were no significant differences in other variables such as mood, quality of life, or overall well‐being, and were less likely to be prescribed antidepressants and antipsychotic medications [4, 21, 34, 35, 42].

### 3.2 Needs of Individuals Living Alone With Dementia

Individuals living alone with dementia have more unmet needs than those living with others in certain categories [24, 33, 36, 42]. Specific needs identified include home care, self‐care, daytime activities including ADLs and IADLs, preventing accidental self‐harm, support for psychological distress, aid for hearing and eyesight challenges, medication management, keeping routines constant and familiar, diagnostic investigation advocacy and support, functional support, and social support [9, 18, 22, 24, 31, 33, 36, 37, 38, 39, 40]. Provider services utilized by individuals living alone with dementia varied, with one study reporting that outpatient services, including psychiatrist and general practitioner appointments, were utilized most frequently [36], and another study reported utilized visits to the neurologist, psychiatrist, and hospital were fewer than individuals with dementia living with others [9]. Additionally, these individuals need support to maintain or sustain their current living arrangements. This can be accomplished through keeping routines constant and familiar, as they often express a desire to continue living independently at home or they may not be able to afford to move to assisted living as it is often more expensive than remaining at home [24, 37].

### 3.3 Perspectives of Individuals Living Alone With Dementia

Ten articles sought to understand the unique perspective of those living alone with dementia and focused on the individual's experiences with diagnosis, *n* = 1 [7], support after diagnosis, *n* = 1 [19], social networks and befriending, *n* = 3 [29, 32, 41], life alone, *n* = 2 [19, 26], their perspective on the use of care and support services, *n* = 1 [28], and their perspective of their living environment at home and in their neighborhood, *n* = 2 [27, 30]. Individuals living alone with dementia experience initial relief and then distress after a dementia diagnosis [7, 20]. Uncertainty, responsibility for care management after diagnosis, loneliness, boredom, maintaining social connections for support, an unclear knowledge of services that are utilized and services that are available in the community, purpose, identity, risk, and support strategies are all identified themes from the research [7, 19, 20, 26, 27, 28, 29, 30, 32, 41]. Individuals living alone with dementia face many uncertainties in the unpredictable trajectory of dementia, fluctuation in cognition, and knowledge of how to continue to live independently [7, 20]. In addition, they may perceive the need for formal support differently, as well as their perception of needs and their own health [28].

Individuals living alone with dementia perceive social connections to include interactions with family, friends, healthcare workers, neighbors, and even minor social acquaintances, such as store workers, which often provide connection and reassurance and decrease loneliness and boredom [26, 27, 29, 30, 32, 41]. There is variation in reporting social support and networks for individuals living alone with dementia [4, 9, 18, 36, 42]. Some individuals living alone with dementia report having more social contact despite smaller social networks [4, 42], some have lower self‐perceived social support [26, 27], and some studies report no significant differences in the number of social networks one has based on living situation [36]. Programs in the community that provide regular companionship and facilitate friendships through volunteers can help satisfy unmet needs and improve social isolation [41]. Individuals living alone with dementia recognize the need for access to public places near their homes and friendly, safe, walkable neighborhoods often provide them the opportunity to get exercise and to look for social interactions with others [30, 32]. Loneliness and boredom may influence the individual's ability to manage life, find purpose, and meaning for the individual living alone with dementia [26, 29]. Decreased ability to communicate, fluctuation in cognition, and worsening cognitive function often brings shame, exhaustion, and anxiety for such individuals, which also inhibits socialization and the individual's ability to feel as if they are thriving independently [20]. Individuals living alone with dementia also recognize the need to have strategies in place to cope with their challenges with cognition, such as using the internet and calendars for orientation, changing communication styles to conceal their changes in cognition, or decreasing social interaction [20].

### 3.4 Services and Interventions Impacting the Individual Living Alone With Dementia

Services and supports include home meal delivery, housekeeping assistance, professional home care, help with medication management, case management, senior centers, adult day care, and community activity groups [9, 17, 24, 32, 36]. Individuals living alone with dementia had more unmet food, self‐care, and medication management needs despite more often utilizing home‐delivered meal services and home care services than individuals living with others [9, 24, 36]. Organizations within the community that provided weekly activities gave individuals living alone with dementia something to look forward to, as well as an opportunity for social interaction [32]. Additionally, the participants often viewed the volunteers and professionals at these activities as friends and felt as if the environment was safe and free of stigma [32]. However, some individuals living alone with dementia who participated in planned activity groups had a negative experience because it was not a personalized approach, and they did not prefer this as a strategy for support or socialization [29].

Barriers to utilization of services and supports include inefficient coordination of services within the community, failure to follow instructions for medical care, patient lack of knowledge of services or supports, lack of specialized services, and desire to remain independent [7, 19, 20, 24, 26, 36]. Lack of specialized services after diagnosis of dementia for individuals living alone can often create a sense of pressure for them as many individuals feel the responsibility for managing their condition so that they are able to have continued independence [7, 19, 20]. Individuals living alone with dementia who are receiving support from the community may not have knowledge of the services they are receiving or why they are needed [26].

## 4 Discussion

The results of this integrative review demonstrate that individuals living alone with dementia have unique characteristics, needs, and perspectives. While these individuals have more social contact and higher functional ability, they have a higher risk of severe loneliness and higher unmet needs than those living with someone. Needs included social services and activities in addition to support in instrumental activities of daily living and activities of daily living as the individual's cognitive function declines. The findings on perspectives of individuals living alone with dementia focus on diagnosis and how their diagnosis impacts their outlook on the future, relationships, social networks, and feelings. Common community‐based services and interventions for individuals living alone with dementia include meals, home care, medication management, and social activities services, but barriers may be present for individuals living alone that may hinder the use of these services and interventions.

### 4.1 Characteristics of Individuals Living Alone With Dementia

Widowed women are at a 20% increased risk of developing dementia, and those with dementia who live alone experience lower income due to the loss of their spouse [44]. The included studies did not examine the differences in racial minorities or environmental factors that contribute to these characteristics, as the majority of participants in studies that included race or ethnicity were White [4, 7, 10, 17, 18, 20, 36, 42]. In addition, individuals living alone with dementia often have a higher functional ability but are at higher risk for severe loneliness in comparison to those living with others [4, 10, 16, 34, 42].

Several gaps are noted in the research findings, including a need to better understand how the cognitive abilities of individuals living alone with dementia impact care transitions in comparison to those living with a care partner or other adult, as the current literature findings show small differences in cognition, and it is unknown how level of cognition impacts care transitions [4, 10, 16, 17, 21, 23]. Among studies reporting significant differences, the approximate difference between the two groups varied by 0.2–0.4 of a point [4, 10, 16, 17, 21, 23]. These statistical differences appeared attributable to a larger sample size in some studies than others [17, 23]. Moreover, education level was not adjusted by some studies, which may further contribute to inconsistent findings [21, 23, 43]. Factors that impact cognition, such as educational level [43], should be considered when reporting cognition test scores. Income and financial status, as well as racial or cultural influence, may impact a timely diagnosis of dementia as well as the care and services received by the individual [45, 46, 47]. Understanding the differences in racial minorities and how environmental factors, such as income and financial status, play a role in the services and supports is needed to better understand this population and to inform care and creation of community services and supports. Addressing these gaps to improve knowledge and understanding of the characteristics of individuals living alone with dementia is imperative to inform clinical practice and the development of community services.

### 4.2 Needs of Individuals Living Alone With Dementia

Individuals living alone with dementia have unique care needs compared to those living with a care partner or other adult [9, 18, 22, 24, 31, 33, 36, 37, 38, 39, 40]. Since the individual does not have someone living with them, there is no one there to give a perspective of how they meet their needs each day and to observe and support how the individual functions and completes IADLs and ADLs. In addition, safety is a greater concern because of risk of accidental self‐harm, money management, and the possibility of being taken advantage of financially, falls, and medication errors such as overdosing, forgetting doses, or taking unnecessary over the counter or discontinued medications [35, 39]. A higher risk for institutionalization, hospitalization, and accidental self‐harm was discovered in individuals living alone with dementia [22, 24, 39, 40]. Most research focuses on the individual's ability to complete ADLs and IADLs and there is limited focus on the individual's social and emotional wellbeing.

Healthy older adults that do not have dementia are found to be more socially active including participating in regular social activities and groups, as well as traveling more frequently [48]. Whereas individuals living alone with dementia often have less social interaction and have greater unmet social needs that may be improved by greater social support and community programming [33].

Gaps in the research include addressing the need to determine barriers to giving equitable care, regardless of their housing situation, evaluation of unmet social needs, as well as provider perspective on differences in managing care for individuals living alone with dementia. Additionally, research is needed to better understand how living situation influences evaluation, diagnosis, and medical management in individuals living alone with dementia [4, 21, 31, 35].

### 4.3 Perspectives of Individuals Living Alone With Dementia

Understanding the perspectives of individuals living alone with dementia is essential to creating community‐based, person‐centered support programs as well as informing care delivered by providers. The literature included in the present review focuses on the individual's perspectives regarding diagnosis, lack of support from providers after diagnosis, relationships, and the individual's outlook on the future [7, 19, 20, 26, 29, 32]. Common themes throughout the literature include uncertainty, continued independence, the desire to feel connected socially, coping strategies, and loneliness and help shed light on aspects of life that are impacted by diagnosis and important to the individual living alone with dementia [7, 20, 26, 27, 28, 29, 30, 39, 41]. Despite the desire for individuals living alone with dementia to feel connected socially, there is variation in the data on how social networks and self‐perceived social support vary by living situation [4, 9, 18, 36]. Thus, individual perception of social networks and actual social support is unknown in individuals living alone with dementia. Understanding the role that individual perception of social networks and actual social support is important in this population for the development of meaningful community programming that meets the social needs of individuals living alone with dementia. There is limited literature exploring whether available services and programming are meeting the needs of individuals living alone with dementia from their perspective [19].

Understanding the perspectives of individuals living alone with dementia can be utilized to apprise service and support development and provider referrals in the community. Due to the limited research on the perspectives of individuals living alone with dementia, there are many gaps in the research that currently exist. Implications for research include addressing gaps in understanding the perspective of individuals living alone with dementia on emotional risk associated with dependence on services or care in the home [40] (and the different dimensions of loneliness that impact these individuals [29]). There is limited understanding of what community‐based, person‐centered support looks like from the unique perspective of individuals living alone with dementia as well as their personalized needs. Additionally, there are gaps in understanding the perspective of these individuals regarding their current living situation, barriers to support and services, and care transitions both in the home and to other living situations.

### 4.4 Services and Interventions Impacting Individuals Living Alone With Dementia

Services and interventions that are available to individuals living alone with dementia include meal‐delivery services, home care services, medication management, and community activities [9, 24, 25, 29, 32, 36]. Despite these common services being offered in the community, individuals living alone with dementia have more unmet needs than those living with a care partner or other adult [9, 24, 36]. This could indicate that the services available to meet these needs did not provide the right help or not to the necessary extent [36]. Individuals living alone with dementia may also refuse needed services because they may feel that they do not need the services or may be concerned about losing their independence [49]. Social opportunities provided by community‐based programs for individuals living alone with dementia are important for maintaining connections in the community, giving purpose, and providing a stigma‐free, safe environment [32].

Several gaps are identified in the current literature, including the need for a better understanding of personalized care and support after diagnosis to improve quality of life and continued personalized care throughout the progression of dementia [7, 20]. Several of the studies in this review included some information about services and service use, but it was not a focus of the studies [3, 14, 15, 16, 25, 26, 30, 31, 43]. The current literature does not attempt to understand how to best deliver care and services to individuals living alone with dementia to assist with remaining in the community as long as possible and does not evaluate whether these individuals receive the appropriate number of formal services that they need [24, 25]. In addition, there are gaps in understanding how community‐based programs can best identify individuals who have potential unmet needs or how to determine individuals living alone with dementia who are at high risk for receiving an insufficient number of services or supports [25].

## 5 Clinical Implications

Clinical implications of the findings of this review include being aware of the demographics, characteristics, and needs of this population to better support and serve, as well as inform healthcare delivery. Providers must be able to recognize and assess the characteristics of individuals living alone with dementia to better guide personalized care. The perspectives, needs, and services used should be assessed for all individuals living alone. Geriatric assessment tools, including assessments for cognition, functional status, psychological well‐being, social support, and spirituality, should be utilized to establish the baseline of their cognitive, physical, and emotional status with regular reassessment, for example, annually, to identify long‐term trends [16]. Emphasis should be placed on establishing baseline cognitive and functional screening in individuals living alone with dementia to gauge the need for services, support, and care assistance and to personalize resources for the individual. Ongoing screening should be conducted by providers to track longitudinal changes and refer to services, supports, and resources as the individual's needs change. The needs of individuals living alone with dementia should be assessed and addressed so that proper, personalized referrals to community‐based resources that offer services and support can be made to assist in meeting these individualized needs. Additionally, providers that care for individuals living alone with dementia should be familiar with resources, supports, and services within the community as well as appropriate tools to identify unmet needs. Providers must also be aware that individuals living alone with dementia may refuse services, but these services should still be offered to the individual as their needs change [49]. Programs, such as the Guiding an Improved Dementia Experience (GUIDE) Model in the United States, offer additional services, resources, and supports to individuals living with all types of dementia [50]. Individuals living alone with dementia who are enrolled in Medicare can directly benefit from the GUIDE Model programs as the services are covered and include care coordination, services, resources, and supports to help the individual continue to live at home as long as possible [50].

Utilization of a comprehensive geriatric assessment tool at baseline and annually is necessary to properly assess cognition, ability to complete ADLs, social health, safety, and emotional well‐being, as well as identify individual needs and perspectives in individuals living alone [16]. Individuals living alone are less likely to recognize cognitive changes, and their providers are often less likely to recognize the cognitive changes that may lead to a diagnosis of dementia [16]. Thus, it is imperative to routinely screen for cognitive changes and safety concerns in all individuals living alone who are older adults [16]. In addition, providers should be aware of the risks involved with individuals living alone with dementia and ensure that safety, psychological, and health assessments are completed to address any needs [4, 42]. Providers may also use this assessment to evaluate services and resources that are currently used to meet the identified needs of the patient. Provider follow‐up and care utilization needs screening, and the ability to connect to resources and support is crucial to best meet the needs of and support individuals living alone with dementia [4, 9, 24, 33]. Finally, the perspectives of individuals living alone with dementia should be addressed and utilized to inform care while continuing to support independence. Providers could engage older adults living alone with dementia in care planning to devise a plan of care that is relevant to older adults living alone with dementia. This will ensure that they will be more likely to undertake the services offered to them to achieve their goals and prevent a waste of resources, time, and money.

## 6 Strengths and Weaknesses

The strengths of this literature review include the use of PRISMA review (see Table S3), quality appraisal of the articles utilizing the JBI Critical Appraisal Tools [13, 14, 15], and it is conceptually unique as there is no other review that examines the posed research questions. Weaknesses of the review include the variation in the types of studies as well as the timeframes in which the studies were conducted, which made it tremendously difficult to compare and draw conclusions. Single screening and data extraction were completed by the first author. The methodological quality of existing studies limits the findings, and precise measurements for older adults living alone with dementia are critical to ensure the scientific rigor of findings in future research. In addition, this review focuses on the characteristics, needs, perspectives, and services of individuals living alone with dementia and not those living with a care partner, and it is possible that some of the findings and recommendations might also apply to individuals living with a care partner with dementia.

## 7 Conclusion

While there is research evaluating the characteristics of individuals living alone with dementia, there is limited evidence examining the needs, perspectives, and impact of services available. Understanding the differences in this population is imperative to informing care and developing meaningful community‐based programs that offer accessible support and services. Geriatric assessment tools must be incorporated into provider visits to screen, identify, and properly support individuals living alone with dementia. In addition, providers and staff should have appropriate training to administer screening tools correctly. Opportunities to better understand the perspectives and priorities for care of individuals living alone with dementia, as well as how available community‐based services provide essential support to these individuals, will help shape future provider education, community‐based programming, and policy.

## Author Contributions

**Sara J. Crance:** conceptualization, data curation, formal analysis, investigation, methodology, writing–original draft, writing–review and editing. **Fang Yu:** conceptualization, formal analysis; investigation, methodology, supervision, writing–original draft, writing–review and editing.

## Conflicts of Interest

The authors declare no conflicts of interest.

## Transparency Statement

The lead author Sara J. Crance affirms that this manuscript is an honest, accurate, and transparent account of the study being reported; that no important aspects of the study have been omitted; and that any discrepancies from the study as planned (and, if relevant, registered) have been explained.

## Supporting information

Supporting information.

## Data Availability

The data that support the findings of this study are available from the corresponding author upon reasonable request.
