# Supplementary material for: Characteristics, Needs, and Perspectives of Individuals Living Alone With Dementia: An Integrative Review
Source: Health Sci Rep. 2025 Jan 23;8(1):e70348. doi: 10.1002/hsr2.70348 (PMC11757279; doi:10.1002/hsr2.70348)
Supplement: Supplementary file 1 — Supporting information. [file HSR2-8-e70348-s001.docx]

**Supplementary Material**

**Table 1**

*Description of search strings utilized with each search engine.*

| **Search Engine** | **Search String** |
| --- | --- |
| PubMed | ((dementia[MeSH Terms]) OR (Alzheimer’s Disease[MeSH Terms])) AND (living alone[Title/Abstract] OR solitary living[Title/Abstract] OR community dwelling[Title/Abstract]) |
| CINAHL | ((MH alzheimer's disease OR MH demenia) OR TI (alzheimer's or alzheimers or alzheimer or alzheimer's OR dementia)) AND (AB ((Living OR dwelling) AND (alone OR community OR solitary OR independent)) OR TI ((Living OR dwelling) AND (alone OR community OR solitary OR independent))) |
| PsycInfo | subject(dementia) AND subject(Alzheimer*) AND subject(living alone) |

**Table 2***Description of study, measures, main findings, and quality score for selected articles.*

| **Author, Year, Country** | **Design** | **Sample Inclusion**  **Exclusion** | **Measures** | **Main Findings** | **Quality** |
| --- | --- | --- | --- | --- | --- |
| 41.Andrew et al., (2022)  United Kingdom - Scotland | Qualitative | **Sample:**  **n**=3, female, n=2, male, n=1, aged 69, 79, and 90, lived alone 100%  **Inclusion:**  Diagnosis of dementia, capacity to participate in research, met volunteer befriender through a befriender organization, unpaid befriender, and befriender service, relationship-centered, lived in the community, in befriender relationship for over 1 month, ability to communicate in English, and willing to be audio recorded | Case study – narrative interviews integrating storytelling exploring topics of befriending, everyday experiences, other relationships or social networks, and life story. | Themes identified:  -Unmet needs satisfied by befriending and wishes for this type of relationship  -Befriending as a facilitated relationship including the impact of memory loss on befriending experiences  -Befriending as a human response to contingent and existential limitations | JBI Critical Appraisal Qualitative Study-  Score of 10/10 |
| 31. Cermaková et al., (2017)  Sweden | Cohort | **Sample:**  n=26,163, women 62%, mean age of 80 years, lived alone 46%.  **Inclusion:**  Diagnosis of AD or mixed dementia  **Exclusion:**  Duplicate cases (n=258), diagnosis other than AD or mixed dementia (n=29,876), individuals not living in ordinary housing or missing data (n=2,013). | Diagnosis and medical management including:  Basic diagnostic work-up: MMSE, Clock test, Blood test, CT  Extended diagnostic work-up: MRI, LP, Neuropsychological testing  Prescription drugs | Living alone was associated with:  -Lower use of imagining and biomarker test for diagnosis  -Less use of ChEIs and memantine  -More frequent use of antidepressants, cardiovascular drugs, anxiolytics, antipsychotics, and hypnotics and sedatives | JBI  Critical Appraisal Cohort Study – Score of 8/11  Deficiency noted in follow-up assessment as this was a cross-sectional cohort study that did not include follow-up with the cohort. |
| 42.Clare et al., (2024)  United Kingdom | Cohort | **Sample:**  T1, n=1,525, T2, n=1,165, T3, n=840, individuals living alone, n=281, individuals living with others, n=1,244  **Inclusion:**  Clinical diagnosis of dementia, MMSE score of 15 or above, ability to verbally communicate in English  **Exclusion:**  Co-morbid terminal illness, inability to provide informed consent, and any known potential significant risk for researchers to visit the home | Characteristics and demographic information, use of health and social care services, use of assistive technology, MMSE, Addenbrooke’s Cognitive Examination-III, Charlson Comorbidity Index, Functional Activities Scale, GDS, De Jong-Gierveld Loneliness Scale, Lubben Social Network Scale, Stigma Impact Scale, Quality of Life in AD Scale, Satisfaction with Life Scale, and World Health Organization-Five Well-Being Index. | Individuals living alone were:  -More likely to be female  -Older on average (over the age of 80)  -Widowed  -Had more co-morbid conditions at baseline  -Rated their health less positively  -Scored higher on cognitive tests and self-reported functional ability  -Decline in cognition was slower over time  -Poorer scores for quality of life, satisfaction with life, and well-being  -More social contact  -Experienced greater loneliness and depression  -Used home care services more  -Used assistive technology more at baseline  -Less likely to visit the nurse at the GP and more likely to have home nurse visits  -Had a increased number of consults with occupational therapists over time  -Had a higher risk of moving into residential or nursing homes over time | JBI Critical Appraisal Cohort Study – Score of 11/11 |
| 4. Clare et al., (2020) United Kingdom | Cross-sectional | **Sample:**  n= 1541 total Individuals living alone with dementia n=285 (18.5%) of which 51 identified as having little or no support (3%)  **Inclusion:**  Clinical diagnosis of dementia, MMSE score of 15 or above, residing in the community.  **Exclusion:**  Inability to provide informed consent, terminal illness, potential risk to researcher for home visits. | Characteristics and demographic information.  Cognitive function – MMSE and Addenbrooke’s Cognitive Examination-III.  Psychological characteristics – Rosenberg Self-Esteem Scale, Generalized Self-Efficacy Scale, Life Orientation Test-Revised, De Jong Gierveld Loneliness Scale, Stigma Impact Scale for people with dementia, and Representations and Adjustment to Dementia Index.  Lubben Social Network Scale and Cultural Capital and Social Exclusion Survey.  Quality of Life in Alzheimer’s Disease Scale, Satisfaction with Life Scale, and WHO Five Well-Being Index.  Use of services. | Individuals living alone were:  -Older on average  -More likely to be female  -More likely to have higher cognitive ability and self-reported functional ability  -More social contact  -Lonelier  -Expressed less satisfaction with life  -Used home care services and equipment more.  -There were no differences in symptoms, mood, quality of life, or well-being. | JBI Critical Appraisal Cross-Sectional Study – Score of 8/8 |
| 29. Duane et al., 2013  Australia | Qualitative | **Sample:** n=19  Female, n=13, aged between 79 and 93 years and male, n=6, aged between 69 and 91 years, that had been living alone an average of 14 years.  **Inclusion:**  Diagnosis of dementia or cognitive impairment, current user of chosen community service, age 65 or older, lives alone  **Exclusion:**  Extreme ill health, advanced cognitive impairment or dementia, or experiencing psychosocial stress | Conversational style interviews exploring social supports, practical supports, interests, social activities, and desired supports. Field notes were obtained and reviewed with the participants. | Themes:  -Life alone  -Social contact and support networks  -Purpose and identity in older age  -Risk  - Support strategies | JBI Critical Appraisal Qualitative Study- Score of 8/10  Deficiency in researcher cultural or theoretical statement and lack of addressing influence of researcher on research and vice-versa. |
| 9. Eichler et al., 2016  Germany | Cross-sectional | **Sample:** n=511  Female, n=303 (59.3%), living alone, n=260 (50.9%), mean age of 80.3 years  **Inclusion:**  Aged 70 or older, community-dwelling, screened positive for dementia, DemTect score <9, ability to sign informed consent or named a caregiver to sign on their behalf. | Socio-demographic characteristics, MMSE, GDS, Bayer Activities of Daily Living Scale, Quality of Life-AD, Social Support Questionnaire (FSozu), yes/no questions on health care and nursing care utilization over the previous 12-month period. | - Approximately half of the community dwelling PWD live alone and approximately 9% of those individuals did not identify an informal caregiver  -PWD living alone did not seem to be at an increased health risk.  -The majority (70%) if PWD living alone were widowed females.  -PWD living alone utilize health care offers less frequently but utilize professional services more often than those not living alone.  -Self-perceived social support was significantly lower in PWD living alone | JBI Critical Appraisal Cross-Sectional Study – Score of 7/8  Deficiency in clearly defined inclusion criteria for sample. |
| 22.Ebly et al., 1999  Canada | Cohort | **Sample**: n=317  **Inclusion:**  Diagnosis of dementia, caregiver agreed to be interviewed.  **Exclusion:**  Living in a long-term care facility | Age, educational level, dementia severity, functional status, living arrangement, and the Modified Mini Mental State examination for participants.  ZBS and the Dementia Behavior Disturbance Scale were measured in caregivers.  Short-term institutionalization rates measured at follow-up and intent to institutionalize | Significant predictors for institutionalization included functional status, location in which the participant lived, and caregiver relationship other than spouse or child.  Intent was predicted by living alone, living arrangements, behavioral problems, and whether the caregiver had considered institutionalization. | JBI  Critical Appraisal Cohort Study – Score of 10/11  Deficiency in noting whether the exposure was measured in a valid and reliable way (unclear). |
| 18. Ennis et al., (2014)  United States | Cohort | **Sample:** n=2,636,  individuals living alone with dementia n= 61 (6.4%), individuals living with others with dementia n=115 (14.6%)  **Inclusion:**  Enrolled in the Adult Changes in Thought (ACT) cohort study, aged 65 or older, not residing in a nursing home.  **Exclusion:**  Left the health plan during the study, periods that were longer than 2.5 years or missed follow-up visits, missing living situation data. | Living situation, presence of dementia, cognitive screening using the Cognitive Abilities Screening Instrument, comorbidity burden measured by the RxRisk Score, and hospitalization. | Dementia did not modify any observed associations of individuals living alone and hospitalization for all causes and ambulatory care sensitive conditions | JBI Critical Appraisal Cohort Study – Score of 10/11 Deficiency in noting whether the outcome was measured in a valid and reliable way (unclear). |
| 39. Gilmour, 2004  United Kingdom - Northern Ireland | Qualitative | **Sample:** n=10  Men living with dementia, n=2, women living with dementia, n= 8, with an average age of 83.3 years  Family members, n=12, general practitioners, n=6, district nurses, n=4, social work assistants, n=2, social workers, n=4.  **Inclusion:**  Diagnosis of dementia and living alone in the community | Open question interviews with the individual living with dementia focusing on daily circumstances, health, awareness of diagnosis, contentment, involvement of family and friends, concerns and risks, current care, views on the future. Family, friends, care givers, and providers were also interviewed if they were available. | -Professionals emphasized physical safety while individuals living with dementia and their families focused on maintaining self-identity and interpersonal relationships. -The main areas of risk that emerged from the study:  -heating and cooking  -falling  -getting lost  -managing money  -None of the people with dementia were concerned about the practicalities of risk, but 2 of the participants indicated that services were unwanted and unnecessary. | JBI Critical Appraisal Qualitative Study- Score of 8/10  Deficiency in researcher cultural or theoretical statement and lack of addressing influence of researcher on research and vice-versa. |
| 40. Gilmour et al., 2003  United Kingdom - Northern Ireland | Qualitative | **Sample:** n=10  Men n=2, women n=8, ranging in age from 74 to 93, known to social services n=9.  **Inclusion:** Diagnosis of dementia, living alone in the community, agreement to participate. | Semi-structured interviews with the individual living alone with dementia. Separate interviews with the individual’s next of kin.  Barthel Index to assess ADLs. | -No major incidents of harm reported  -Two preventative factors that minimize harm: concern and observation of others known to the person with dementia and level of contact by others in the day-to-day life  -The main areas of risk: heating and cooking, falling, getting lost and managing money | JBI Critical Appraisal Qualitative Study- Score of 8/10  Deficiency in researcher cultural or theoretical statement and lack of addressing influence of researcher on research and vice-versa. |
| 37. Heaton et al.,2021  United Kingdom | Qualitative | **Sample:** n= 24 dyads that consisted of an individual living alone with dementia and a relative or friend that provides support  Female n=21, male n=3, age range of 69-91 years, MMSE score range of 19-29.  **Inclusion:**  Diagnosis of AD, vascular dementia or mixed dementia and living alone in the community with a friend or relative that provides support. | Secondary data analysis from the Memory Impairment and Dementia Study interviews conducted with the dyads. | Themes identified:  -Future outlook  -Care and living arrangements  -Holding on  -Anticipatory acts | JBI Critical Appraisal Qualitative Study- Score of 8/10  Deficiency in researcher cultural or theoretical statement and lack of addressing influence of researcher on research and vice-versa. |
| 28. Illiger et al., 2021  Germany | Qualitative | **Sample:** n=12  Women, n=10, men, n=2  **Inclusion:**  65 years of age or older, medically diagnosed with dementia | Biographical narrative interviews | Themes identified:  -Mindsets and beliefs based on past care experiences  -Subjectively perceived resources  -Subjective health  -Subjective need | JBI Critical Appraisal Qualitative Study- Score of 9/10  Deficiency in researcher cultural or theoretical statement. |
| 16. Lehmann et al., 2010  United States | Cross-sectional | **Sample:** n= 349: individuals living alone with dementia n=97, individuals living with dementia with others n=252  **Inclusion:**  65 years of age or older, score of <24 on the MMSE, or decline of at least 4 points on the MMSE over 2 administrations, classified as having dementia. | Cognitive impairment: MMSE  Functional impairment: standardized ADL index and instrumental ADL.  Psychiatric symptoms: NPI, Behavior Symptom Rating Scale, agitation rated by a knowledgeable informant | In individuals living alone with dementia:  -mean MMSE scores were significantly higher  -fewer ADL and IADL impairments  -had significantly fewer helpers  Individuals living with dementia with others were:  -more likely to be agitated  -more likely to be rated as having fair or poor mental health compared to individuals living alone with dementia  -No significant difference in physical health ratings by the knowledgeable informants for either group.  -Knowledgeable informants and physicians are less likely to recognize cognitive impairment if the individual lives alone. | JBI Critical Appraisal Cross-Sectional Study – Score of 8/8 |
| 30. Lloyd & Stirling, 2014 Australia | Qualitative | **Sample:** n=7  Age range between 48-85 years.  **Inclusion:**  Diagnosis of early to moderate dementia, living alone in the community | Semi-structured interviews in the participants’ homes with open-ended questions | Themes identified:  -Access to public space  -Social distance and proximity  -Changing meanings of space and objects  -Imaginative co-presence (television programming, items in their homes, remembering individuals or places, and their home being a source of comfort and joy) | JBI Critical Appraisal Qualitative Study- Score of 8/10  Deficiency in researcher cultural or theoretical statement and lack of addressing influence of researcher on research and vice-versa. |
| 33. Meaney at al., 2005 Ireland | Cross-sectional | **Sample:** n=82  Female n=55, male n=27, mean age of 76 years, mean MMSE score of 15.9  **Inclusion:**  Diagnosis of dementia, living in the community, referral to psychiatry for assessment. | MMSE, Care Needs Assessment Pack for Dementia (CareNap-D) | Individuals living alone with dementia had a higher rate of unmet needs across all domains:  -Behavior and mental state  -Self-care and toileting  -Social interaction  -Health and mobility  -Community living  -House care  Individuals living alone with dementia had significantly more unmet needs in the following domains:  -Thinking/memory  -Housework  -Community living | JBI Critical Appraisal Cross-Sectional Study – Score of 8/8 |
| 36. Miranda-Castillo et al., 2010  United Kingdom | Cross-sectional | **Sample:** n=152  PWD living at home, n=128 informal caregivers from health and social services; n=50 PWD living alone  **Inclusion:**  Aged 60 years or older, diagnosis of dementia, living at home. | Camberwell Assessment of Need for the Elderly (CANE), MMSE, NPI, Physical Self-maintenance Scale (PSMS), Quality of Life in Alzheimer’s Disease (QoL-AD), Practitioner Assessment of Network Typology (PANT), Client Service Receipt Inventory (CSRI), Hospital Anxiety and Depression Scale (HADS), and ZBI | PWD living alone had significantly more unmet needs than PWD living with others, especially in:  -Caring for their home  -Self-care  -Daytime activities  -Company  -Psychological distress  -Eyesight or hearing  -Accidental self-harm | JBI Critical Appraisal Cross-Sectional Study – Score of 8/8 |
| 23. Nourhashemi et al., 2005  France | Cross-sectional | **Sample:** n=677  living alone n=186 and living with others n=491  **Inclusion:**  Diagnosis of AD, lived in the community, and were looked after by informal caregiver. | MMSE, NPI, Mini-Nutritional Assessment (MNA), ZBI, current function and mobility in ADLs and instrumental activities of daily living (IADL) | Individuals living alone with dementia were:  -Significantly older  -Higher percentage of women  -At an increased risk of malnutrition  -More likely to have a lower income  -Made greater use of health services  At one-year follow-up, individuals living with others:  -Had a significantly higher mortality rate than those living alone. | JBI Critical Appraisal Cross-Sectional Study – Score of 8/8 |
| 27. Odzakovic et al., 2020 Sweden | Qualitative | **Sample:** n=14  Men n=11, women n=3, ranging in age from 62-87 years  **Inclusion:**  Diagnosis of dementia, living in the community | Walking interviews throughout the participant’s neighborhood | Themes identified:  -Life narratives embedded within the neighborhood  -Support of selfhood and wellbeing  -Neighborhood as an immediate social context  -Connections to nature | JBI Critical Appraisal Qualitative Study- Score of 8/10  Deficiency in researcher cultural or theoretical statement and lack of addressing influence of researcher on research and vice-versa. |
| 32. Odzakovic et al., 2021  England, Scotland, and Sweden | Qualitative | **Sample:** n=14  Women n=11 and men n=3, ranging in age from 62-88 years  **Inclusion:**  Diagnosis of dementia, living alone, and living in a one-person household | Walking interviews, in-home interviews, home tours, and social network mapping. | Themes revealed:  -Making the effort to stay connected  -Befriending by organizations and facilitated friendships  -Quiet neighborhood atmosphere  -Changing social connections  The study determined that individuals living alone with dementia are active agents who took control to find and maintain relationships and social networks in the neighborhood. | JBI Critical Appraisal Qualitative Study- Score of 8/10 Deficiency in researcher cultural or theoretical statement and lack of addressing influence of researcher on research and vice-versa. |
| 19. Portacolone, 2018  United States | Qualitative | **Sample:** n=1  Female, living alone with dementia, aged 79**.** | Eight ethnographic interviews with one individual that took place between 2014-2017 | A diagnosis of dementia in the United States does not lead to more specialized supports to allow individuals living alone with dementia to remain in their homes (in the community, through insurance, and through state or federal programs) | JBI Critical Appraisal Qualitative Study- Score of 7/10  Deficiency in researcher cultural or theoretical statement, lack of addressing influence of researcher on research and vice-versa, and unclear about evidence of ethical approval. |
| 7. Portacolone et al., 2018  United States | Qualitative | **Sample:** n=29  Individuals living alone diagnosed with AD n=13 and individuals living alone diagnosed with MCI n=16  **Inclusion:**  Living alone, age≥65, medical diagnosis of AD or MCI, ability to provide consent. | Ethnographic interviews and participant observation | Themes included:  -Relief (positive)  -Distress  -Ambiguous recollections  -Not knowing what to do | JBI Critical Appraisal Qualitative Study- Score of 9/10  Deficiency in researcher cultural or theoretical statement. |
| 20. Portacolone et al., 2019  United States | Qualitative | **Sample:** n=12  Women n=10, men n=2, mean age of 79, diagnosis of AD n=6, diagnosis of MCI n=6, non-Latino white n=11  **Inclusion:**  Age ≥65, medical diagnosis of AD or MCI, living alone, ability to provide consent. | Ethnographic interviews and participant observation in their homes. | Themes included:  -Description of the distress stemming from the uncertainty and unpredictable course  -Tendency of participants to feel responsible for managing their cognitive impairment  -Pressures stemming from the lack of appropriate services to support independent living | JBI Critical Appraisal Qualitative Study- Score of 9/10  Deficiency in researcher cultural or theoretical statement. |
| 10. Prescop et al., 1999  United States | Cross-sectional | **Sample:** n=109  Individuals living alone with dementia n=38 and individuals living with others n=71.  **Inclusion:**  Aged 65 years or older, residing in the community, fluent in English, with at least a 6th grade education. | Global cognitive scale (general mental status test), MMSE, Consortium to Establish a Registry for Alzheimer’s Disease (CERAD) neuropsychological panel, Clinical Dementia Rating (CDR) scale, Caregiver Burden Scale, Zung Depression Scale | Individuals living alone with dementia were:  -More likely to be women  -To have dementia of shorter duration and lesser severity  -Lesser functional impairment | JBI Critical Appraisal Cross-Sectional Study – Score of 7/8  Deficiency as strategies to deal with confounding factors were unclear. |
| 38. Read et al., 2021  United Kingdom | Cohort | **Sample:** n=234  Female n=122, mean age 78.4, did not have a partner n=93 (40%)  **Inclusion:**  Aged 50 or older, living in the community at baseline, having dementia or AD.  **Exclusion:**  Living in care homes at baseline | Unmet need and functional limitations– measurement of ADLs, IADLs, and mobility  Socio-demographic factors based on National Statistics Socio-economic Classification (NS-SEC) | -Unmet need was associated with not have a partner, lower wealth, and not being a homeowner  -Functional limitations were associated with older age, not having a partner, and lower socioeconomic status. | JBI Critical Appraisal Cohort Study – Score of 9/11 Deficiency in identifying confounding factors and strategies to deal with confounding factors (unclear). |
| 24. Soto et al., 2015  France | Cohort | **Sample:** n=1,131  Living alone n=348, living with others n=783  **Inclusion:**  Diagnosis of AD, ambulatory, community-dwelling individuals | Socio-demographic factors, comorbidities at baseline, MMSE, physical function based on the AD Cooperative Study -Activities of Daily Living (ADCS-ADL), hospitalization, nursing home admission, death, and weight loss. | Living alone with AD:  -Increase the risk of hospitalization  -Twice the risk of being institutionalized  -Does not increase the risk of mortality or weight loss over 2 years of follow-up compared to individuals living at home with others. | JBI Critical Appraisal Cohort Study – Score of 11/11 |
| 26. Svanström and Sundler, 2015  Sweden | Qualitative | **Sample:** n= 6  Aged between 80-90 years old.  **Inclusion:**  Lived alone in their home, diagnosis of dementia. | Multiple narrative interviews focusing on a reflective lifeworld approach. | Themes included:  -Feelings of loneliness  -Only a vague knowledge of the home care services that they were receiving and why they were receiving them  -Longing for other people that have time to talk and listen  -Feelings of boredom | JBI Critical Appraisal Qualitative Study- Score of 10/10 |
| 21. Tuokko et al., 1999  Canada | Cross-sectional | **Sample:**  Study 1: n= 177  Living alone n=70, living with others n=107  Study 2: n=152  Living alone n=82, living with spouses n=70  **Inclusion:**  Study 1: Living at home in the community, exhibiting possible of probable AD, fluent in English.  Study 2: Diagnosed with AD, living in the community. | Modified Mini-Mental State (3MS) Examination, MMSE, measurement of service utilization, Global Assessment of Functioning scores (GAF), risk behavior checklist, socio-demographic information, measurement of informal social supports | Individuals living alone with dementia:  -Utilized homemaker and home-delivered meal services significantly more frequently  -Were older than those living with a spouse  -Most were woman  -Were viewed as being at risk in the areas of nutrition, money management, medication management, hygiene, fire, and falls.  -Age of onset of dementia was older | JBI Critical Appraisal Cross-Sectional Study – Score of 6/8  Deficiency in detailed description of study subjects and setting and unclear if strategies to deal with confounding factors were addressed. |
| 34. Victor et al., 2019  United Kingdom | Cross-sectional | **Sample:** n=1547  Men n=871, women n=676, living alone n=285  **Inclusion:**  Clinical diagnosis of dementia (any sub-type), mild to moderate stage of dementia (determined by MMSE score of 15 or above), living in the community.  **Exclusion:**  Comorbid terminal illness, inability to provide informed consent, and any known potential for home visits to pose a risk to researchers | De Jong Gierveld Loneliness Scale, Geriatric Depression Scale, Lubben Social Network Scale Satisfaction with Life Scale, WHO-Five Well-Being Index, Quality of Life in Alzheimer’s Disease Scale (QoL-AD), MMSE | -Loneliness was significantly associated with increased age, living alone, widowhood, depression and isolation, and with lower wellbeing, quality of life and life satisfaction  -Individuals living alone Higher relative risk of experiencing severe loneliness | JBI Critical Appraisal Cross-Sectional Study – Score of 8/8 |
| 25. Wattmo et al., 2014  Sweden | Cohort | **Sample:**  n=1,021  Living alone n=355 and living with a family member n=666  **Inclusion:**  Mild to moderate AD (MMSE score 10-26), living in their own home with or without home-help services at the time of AD diagnosis, have a responsible caregiver, and to be assessable using the MMSE at the start of ChEI therapy**.**  **Exclusion:**  Other causes of dementia | MMSE, Instrumental Activities of Daily Living Scale, Physical Self-Maintenance Scale (PSMS), use of home-help services, nursing home placement | Individuals living alone:  -Were predominantly female  -Used more antidepressant and antipsychotic medications but less lipid lowering agents  -Were significantly older at the onset of AD and start of ChEI treatment.  -ADL capacity was more impaired  -Used more medications at baseline.  -Lower IADL ability and more medications were independent predictors of usage of home-help services.  -More impaired baseline IADL and faster IADL deterioration were predictors of nursing home admission.  -Cognitive ability was not significantly associated with use of community-based services. | JBI Critical Appraisal Cohort Study – Score of 11/11 |
| 17. Webber et al., 1994  United States | Cross-sectional | **Sample: n=2,505**  Individuals living alone n=479 and individuals living with others n=2,026  **Inclusion:**  Clinically diagnosed with probable or possible AD, community dwelling.  **Exclusion:**  Persons diagnosed as having dementia due to multiple infarcts, Parkinson’s disease, Pick’s disease, and alcohol abuse. Reside in nursing homes. | MMSE, Blessed Roth Dementia Rating Scale, socio-demographic information, service utilization, health care service use, and community care service use. | Individuals living alone with dementia are:  -Significantly older  -Female  -More likely to receive SSI and MediCal  -Have had the disease for significantly less time and are less cognitively impaired than those living with others.  -Living arrangement is a significant predictor of service utilization and individuals living alone were less likely to use medical services such as physicians and hospitals and more likely to use services such as homemaker and meal delivery services.  -Overall, individuals living alone were more likely to use no services than those living with others. | JBI Critical Appraisal Cross-Sectional Study – Score of 7/8  Deficiency noted as unclear if strategies to deal with confounding factors were addressed |
| 35. Zafeiridi et al., 2020  United Kingdom - Northern Ireland | Cohort | **Sample:** n=25,418  Mean age 77.30 years, female 65%, lived alone n=8,828 and lived with another person n=12,200. The remaining n=4,390 transitioned to a care home and were excluded from the final analysis  **Inclusion:**  Dementia diagnosis, living in the community, using a dementia management medication**.**  **Exclusion:**  Transition to a care home | Socio-demographic information, type of dementia medication, use of comorbidity medication | Individuals living alone with dementia were more likely:  -To be widowed females --Younger than individuals living with another person  -Less odds of being prescribed antidepressants and medications for comorbidities.  -More likely to be prescribed Donepezil. | JBI Critical Appraisal Cohort Study – Score of 11/11 |

Key: **AD**- Alzheimer’s Disease, **MMSE**- Mini Mental Status Exam, **LP**- lumbar puncture, **ChEIs** – cholinesterase inhibitor, **WHO-** World Health Organization, **GDS**- Geriatric Depression Scale, **ZBS –** Zarit Burden Scale, **PWD** – people with dementia, **ADLs**- activities of daily living, **NPI-** Neuropsychiatric Inventory, **MCI** – mild cognitive impairment

**Table 3**

**PRISMA Checklist**

| **Section and Topic** | **Item #** | **Checklist item** | **Location where item is reported** |
| --- | --- | --- | --- |
| **TITLE** |  |  |  |
| Title | 1 | Identify the report as a systematic review. | Integrative review |
| **ABSTRACT** |  |  |  |
| Abstract | 2 | See the PRISMA 2020 for Abstracts checklist. |  |
| **INTRODUCTION** |  |  |  |
| Rationale | 3 | Describe the rationale for the review in the context of existing knowledge. | Introduction |
| Objectives | 4 | Provide an explicit statement of the objective(s) or question(s) the review addresses. | Introduction |
| **METHODS** |  |  |  |
| Eligibility criteria | 5 | Specify the inclusion and exclusion criteria for the review and how studies were grouped for the syntheses. | Methods section, paragraph 2 |
| Information sources | 6 | Specify all databases, registers, websites, organisations, reference lists and other sources searched or consulted to identify studies. Specify the date when each source was last searched or consulted. | Methods section, paragraph 1 |
| Search strategy | 7 | Present the full search strategies for all databases, registers and websites, including any filters and limits used. | Methods section, paragraph 1 and Table 2 |
| Selection process | 8 | Specify the methods used to decide whether a study met the inclusion criteria of the review, including how many reviewers screened each record and each report retrieved, whether they worked independently, and if applicable, details of automation tools used in the process. | Methods section, paragraph 1 |
| Data collection process | 9 | Specify the methods used to collect data from reports, including how many reviewers collected data from each report, whether they worked independently, any processes for obtaining or confirming data from study investigators, and if applicable, details of automation tools used in the process. | Results section |
| Data items | 10a | List and define all outcomes for which data were sought. Specify whether all results that were compatible with each outcome domain in each study were sought (e.g. for all measures, time points, analyses), and if not, the methods used to decide which results to collect. | Methods section, paragraph 2 and Figure 1 |
|  | 10b | List and define all other variables for which data were sought (e.g. participant and intervention characteristics, funding sources). Describe any assumptions made about any missing or unclear information. | Methods section, paragraph 2 |
| Study risk of bias assessment | 11 | Specify the methods used to assess risk of bias in the included studies, including details of the tool(s) used, how many reviewers assessed each study and whether they worked independently, and if applicable, details of automation tools used in the process. | --- |
| Effect measures | 12 | Specify for each outcome the effect measure(s) (e.g. risk ratio, mean difference) used in the synthesis or presentation of results. | No synthesis preformed |
| Synthesis methods | 13a | Describe the processes used to decide which studies were eligible for each synthesis (e.g. tabulating the study intervention characteristics and comparing against the planned groups for each synthesis (item #5)). | Methods section, paragraph 2 and Figure 1 |
|  | 13b | Describe any methods required to prepare the data for presentation or synthesis, such as handling of missing summary statistics, or data conversions. | --- |
|  | 13c | Describe any methods used to tabulate or visually display results of individual studies and syntheses. | Methods section, paragraph 1 |
|  | 13d | Describe any methods used to synthesize results and provide a rationale for the choice(s). If meta-analysis was performed, describe the model(s), method(s) to identify the presence and extent of statistical heterogeneity, and software package(s) used. | Not applicable |
|  | 13e | Describe any methods used to explore possible causes of heterogeneity among study results (e.g. subgroup analysis, meta-regression). | Themes used as mentioned in Methods section, paragraph 2 |
|  | 13f | Describe any sensitivity analyses conducted to assess robustness of the synthesized results. | Not applicable |
| Reporting bias assessment | 14 | Describe any methods used to assess risk of bias due to missing results in a synthesis (arising from reporting biases). | Not applicable |
| Certainty assessment | 15 | Describe any methods used to assess certainty (or confidence) in the body of evidence for an outcome. | Not applicable |
| **RESULTS** |  |  |  |
| Study selection | 16a | Describe the results of the search and selection process, from the number of records identified in the search to the number of studies included in the review, ideally using a flow diagram. | Results section, paragraph 1 and Figure 1 |
|  | 16b | Cite studies that might appear to meet the inclusion criteria, but which were excluded, and explain why they were excluded. | Supplementary Material, Table 4 |
| Study characteristics | 17 | Cite each included study and present its characteristics. | Results section |
| Risk of bias in studies | 18 | Present assessments of risk of bias for each included study. | --- |
| Results of individual studies | 19 | For all outcomes, present, for each study: (a) summary statistics for each group (where appropriate) and (b) an effect estimate and its precision (e.g. confidence/credible interval), ideally using structured tables or plots. | Results section |
| Results of syntheses | 20a | For each synthesis, briefly summarise the characteristics and risk of bias among contributing studies. |  |
|  | 20b | Present results of all statistical syntheses conducted. If meta-analysis was done, present for each the summary estimate and its precision (e.g. confidence/credible interval) and measures of statistical heterogeneity. If comparing groups, describe the direction of the effect. | Not applicable |
|  | 20c | Present results of all investigations of possible causes of heterogeneity among study results. | Not applicable |
|  | 20d | Present results of all sensitivity analyses conducted to assess the robustness of the synthesized results. | Not applicable |
| Reporting biases | 21 | Present assessments of risk of bias due to missing results (arising from reporting biases) for each synthesis assessed. | --- |
| Certainty of evidence | 22 | Present assessments of certainty (or confidence) in the body of evidence for each outcome assessed. | --- |
| **DISCUSSION** |  |  |  |
| Discussion | 23a | Provide a general interpretation of the results in the context of other evidence. | Discussion section, paragraph 1 |
|  | 23b | Discuss any limitations of the evidence included in the review. | Strengths and Weaknesses section |
|  | 23c | Discuss any limitations of the review processes used. | Strengths and Weaknesses section |
|  | 23d | Discuss implications of the results for practice, policy, and future research. | Discussion section, paragraph 3,6,8,10 and Clinical Implications section |
| **OTHER INFORMATION** |  |  |  |
| Registration and protocol | 24a | Provide registration information for the review, including register name and registration number, or state that the review was not registered. | Not applicable |
|  | 24b | Indicate where the review protocol can be accessed, or state that a protocol was not prepared. | --- |
|  | 24c | Describe and explain any amendments to information provided at registration or in the protocol. | --- |
| Support | 25 | Describe sources of financial or non-financial support for the review, and the role of the funders or sponsors in the review. | Funding Statement |
| Competing interests | 26 | Declare any competing interests of review authors. | Conflict of Interest statement |
| Availability of data, code and other materials | 27 | Report which of the following are publicly available and where they can be found: template data collection forms; data extracted from included studies; data used for all analyses; analytic code; any other materials used in the review. | Data Availability statement |

**Table 4**

**Records Excluded from Review after Full-Text Assessment**

| **Reason Excluded** | **Citations** |
| --- | --- |
| Wrong Population | Abreu et al., 2019,^1^Allison et al., 2022,^2^ Amjad et al., 2016,^3^Amjad & Roth, 2016,^4^  Amjad et al., 2018,^5^ Bae-Sahaaw et al., 2023,^7^ Bazooband et al., 2023,^8^ Benbow & Kingston, 2016,^9^ Bergeron et al., 2023,^10^ Birt et al., 2023,^11^ Boersma et al., 1997,^12^ Bolt et al., 2022,^13^ Campbell et al., 2023,^15^ Clarke et al., 2018,^16^ Csipke et al., 2021,^18^ Dawson et al., 2015,^20^ de Witt & Ploeg, 2016,^23^ Diaz Ponce & Gove, 2016,^24^ Dufour et al., 2023,^26^ Dufour et al., 2024,^25^ Eichler et al., 2016,^27^ Evans et al., 2020,^28^ Gibson & Richardson, 2017,^29^ Gonçalves-Pereira et al., 2024,^30^ Griffith et al., 2016,^31^Gwyther, 1997,^32^ Hansen et al., 2018,^34^ Harkey, 2014,^35^ Harwood et al., 2000,^37^ Herron & Rosenberg, 2017,^39^ Honjo et al., 2023,^40^ Isik et al., 2018,^41^ Khanassov et al., 2021,^44^ Mansfield et al., 2022,^51^ Matsuko et al., 2024,^52^ Mazurek et al., 2019,^54^ Meija-Arango et al., 2021,^55^ Michalowsky et al., 2024,^56^ Miranda-Castillo et al., 2010,^58^ Nakanishi et al., 2020,^61^ O'Connor et al., 1991,^65^ O'Shea et al., 2020,^66^ Okamura et al., 2019,^67^ Phinney et al., 2007,^69^ Reckrey et al., 2020,^72^ Reckrey et al., 2022,^71^ Sampath et al., 2015, ^73^ Scharf et al., 2024,^74^ Sethuram et al., 2022,^75^ Smith et al., 2007,^76^ Swanwick et al., 1999,^77^ Thoma-Lürken et al., 2018,^79^ Tierney et al., 2022,^80^ Ura et al., 2024,^81^ van der Roest et al., 2009,^82^ von Kutzleben et al., 2012,^83^ Weber et al., 2011,^84^ Yeh et al., 2021,^85^ |
| Did not focus on characteristics, needs, perspectives, or services | Brorsson et al., 2013,^14^ Curnow et al., 2022,^19^ de Witt et al., 2010,^22^ Harris, 2006,^36^ Henderson et al., 2022,^38^ Kerpershoek et al., 2020,^43^ Lee et al., 2022,^47^ Lin et al., 2017,^48^ Mazurek et al., 2019,^53^ Michalowsky et al., 2019,^57^ Newhouse et al., 2001,^62^ Nygård & Starkhammar, 2003,^63^ Nygård & Starkhammar, 2007,^64^ Parker & Fabius, 2022,^68^ Tam et al., 2024,^78^ |
| Book, magazine, editorial, or conference proceeding | Aspinal et al., 2023,^6^ Craig et al., 2023,^17^ de Medeiros et al., 2022,^21^ Han & Radel, 2017,^33^  Keady, 1994,^42^ Kirk at al., 2016,^45^ Kolanowski et al., 2018,^46^ Mahon et al., 2019,^49^ Malloy & McLaughlin, 2010,^50^ Miyamae et al., 2022,^59^ Mukaetova-Ladinska, 2017,^60^ Portacolone & Cohen, 2024 ^70^ |

**Excluded Articles**

1. Abreu W, Tolson D, Jackson GA, Staines H, Costa N. The relationship between frailty, functional dependence, and healthcare needs among community‐dwelling people with moderate to severe dementia. *Health & Social Care in the Community*. 2019;27(3):642-653. doi:10.1111/hsc.12678

2. Allison TA, Gubner JM, Oh A, et al. Meaningful Activities and Sources of Meaning for Community-Dwelling People Living with Dementia. *J Am Med Dir Assoc*. 2022;23(7):1191-1196.e1. doi:10.1016/j.jamda.2021.08.009

3. Amjad H, Carmichael D, Austin AM, Chang CH, Bynum JP. Continuity of Care and Health Care Utilization in Older Adults With Dementia in Fee-for-Service Medicare. *JAMA Intern Med*. 2016;176(9):1371-1378. doi:10.1001/jamainternmed.2016.3553

4. Amjad H, Roth DL, Samus QM, Yasar S, Wolff JL. Potentially Unsafe Activities and Living Conditions of Older Adults with Dementia. *J Am Geriatr Soc*. 2016;64(6):1223-1232. doi:10.1111/jgs.14164

5. Amjad H, Wong SK, Roth DL, et al. Health Services Utilization in Older Adults with Dementia Receiving Care Coordination: The MIND at Home Trial. *Health Serv Res*. 2018;53(1):556-579. doi:10.1111/1475-6773.12647

6. Aspinal F, Willcox A, Murphy D, Sanders T, Brooks J. Living alone with dementia - managing without informal support to contact and navigate services: A mixed methods protocol. *J Lang Technol Comput*. 2023;10(1):194. doi:10.31389/jltc.194

7. Bae-Shaaw YH, Shier V, Sood N, Seabury SA, Joyce G. Potentially Inappropriate Medication Use in Community-Dwelling Older Adults Living with Dementia. *Journal of Alzheimer’s Disease*. 2023;93(2):471-481. doi:10.3233/JAD-221168

8. Bazooband A, Courtney-Pratt H, Tierney L, Doherty K. Engaging in participatory community-based arts: perspectives of people living with dementia. *Health Soc Care Community*. 2023;30:88449. doi:10.1155/2023/3088449.

9. Benbow SM, Kingston P. 'Talking about my experiences … at times disturbing yet positive': Producing narratives with people living with dementia. *Dementia (London)*. 2016;15(5):1034-1052. doi:10.1177/1471301214551845

10. Bergeron CD, Robinson MT, Willis FB, et al. Creating a Dementia Friendly Community in an African American Neighborhood: Perspectives of People Living with Dementia, Care Partners, Stakeholders, and Community Residents. *J Appl Gerontol*. 2023;42(2):280-289. doi:10.1177/07334648221130055

11. Birt L, Charlesworth G, Moniz-Cook E, et al. "The Dynamic Nature of Being a Person": An Ethnographic Study of People Living With Dementia in Their Communities. *Gerontologist*. 2023;63(8):1320-1329. doi:10.1093/geront/gnad022

12.Boersma F, Eefsting JA, van den Brink W, van Tilburg W. Care services for dementia patients: predictors for service utilization. *Int J Geriatr Psychiatry*. 1997;12(11):1119-1126. doi:10.1002/(sici)1099-1166(199711)12:11<1119::aid-gps702>3.0.co;2-h

13. Bolt SR, van der Steen JT, Khemai C, Schols JMGA, Zwakhalen SMG, Meijers JMM. The perspectives of people with dementia on their future, end of life and on being cared for by others: A qualitative study. *J Clin Nurs*. 2022;31(13-14):1738-1752. doi:10.1111/jocn.15644

14. Brorsson A, Ohman A, Cutchin M, Nygård L. Managing critical incidents in grocery shopping by community-living people with Alzheimer's disease. *Scand J Occup Ther*. 2013;20(4):292-301. doi:10.3109/11038128.2012.752031

15. Campbell S, Clark A, Keady J, et al. “I can see what’s going on without being nosey...”: What matters to people living with dementia about home as revealed through visual home tours. *International Journal of Geriatric Psychiatry*. 2023;38(9):1-12. doi:10.1002/gps.5999

16. Clarke CL, Keyes SE, Wilkinson H, et al. ‘I just want to get on with my life’: a mixed-methods study of active management of quality of life in living with dementia. *Ageing and Society*. 2018;38(2):378-402. doi:10.1017/S0144686X16001069

17. Craig S, Mitchell G, Halloran PO, Stark P, Wilson CB. Exploring the experiences of people living with dementia in Dementia Friendly Communities (DFCs) in Northern Ireland: a realist evaluation protocol. *BMC Geriatrics*. 2023;23(1):1-10. doi:10.1186/s12877-023-04090-y

18. Csipke E, Shafayat A, Sprange K, et al. Promoting Independence in Dementia (PRIDE): A Feasibility Randomized Controlled Trial. *Clin Interv Aging*. 2021;16:363-378. Published 2021 Feb 25. doi:10.2147/CIA.S281139

19. Curnow E, Rush R, Gorska S, Forsyth K. Differences in assistive technology installed for people with dementia living at home who have wandering and safety risks [published correction appears in BMC Geriatr. 2022 Mar 1;22(1):172. doi: 10.1186/s12877-021-02616-w]. *BMC Geriatr*. 2021;21(1):613. Published 2021 Oct 30. doi:10.1186/s12877-021-02546-7

20. Dawson A, Bowes A, Kelly F, Velzke K, Ward R. Evidence of what works to support and sustain care at home for people with dementia: a literature review with a systematic approach. *BMC Geriatr*. 2015;15:59. Published 2015 May 13. doi:10.1186/s12877-015-0053-9

21. de Medeiros K, Berlinger N, Girling L. Not Wanting to Lose the Dignity of Risk: On Living Alone with Dementia. *Perspect Biol Med*. 2022;65(2):274-282. doi:10.1353/pbm.2022.0023

22. de Witt L, Ploeg J, Black M. Living alone with dementia: an interpretive phenomenological study with older women. *J Adv Nurs*. 2010;66(8):1698-1707. doi:10.1111/j.1365-2648.2010.05295.x

23. de Witt L, Ploeg J. Caring for older people living alone with dementia: Healthcare professionals' experiences. *Dementia (London)*. 2016;15(2):221-238. doi:10.1177/1471301214523280

24. Diaz Ponce AM, Gove D. A comparative analysis of national care pathways for people with dementia living at home in Europe. *International Journal of Integrated Care*. 2016;16(6):A96. doi:10.5334/ijic.2644

25. Dufour I, Margo-Dermer E, Hudon C, et al. Profiles of healthcare use of persons living with dementia: A population-based cohort study. *Geriatr Gerontol Int*. 2024;24(8):789-796. doi:10.1111/ggi.14930

26. Dufour I, Vedel I, Courteau J, Quesnel-Vallée A. Trajectories of care of community-dwelling people living with dementia: a multidimensional state sequence analysis. *BMC Geriatr*. 2023;23(1):250. Published 2023 Apr 27. doi:10.1186/s12877-023-03926-x

27. Eichler T, Thyrian JR, Hertel J, et al. Unmet Needs of Community-Dwelling Primary Care Patients with Dementia in Germany: Prevalence and Correlates. *J Alzheimers Dis*. 2016;51(3):847-855. doi:10.3233/JAD-150935

28. Evans SC, Atkinson T, Cameron A, et al. Can extra care housing support the changing needs of older people living with dementia?. *Dementia (London)*. 2020;19(5):1492-1508. doi:10.1177/1471301218801743

29. Gibson AK, Richardson VE. Living Alone With Cognitive Impairment. *Am J Alzheimers Dis Other Demen*. 2017;32(1):56-62. doi:10.1177/1533317516673154

30. Gonçalves-Pereira M, Marques MJ, F Alves R, et al. Needs for Care, Service Use and Quality of Life in Dementia: 12-Month Follow-Up of the Actifcare Study in Portugal. *Acta Med Port*. 2024;37(5):355-367. doi:10.20344/amp.20427

31. Griffith LE, Gruneir A, Fisher K, et al. Patterns of health service use in community living older adults with dementia and comorbid conditions: a population-based retrospective cohort study in Ontario, Canada. *BMC Geriatr*. 2016;16(1):177. Published 2016 Oct 26. doi:10.1186/s12877-016-0351-x

32. Gwyther LP. The perspective of the person with Alzheimer disease: which outcomes matter in early to middle stages of dementia? *Alzheimer Dis Assoc Disord*. 1997;11(Suppl 6):18-24.

33. Han A, Radel J. The benefits of a person-centered social program for community-dwelling people with dementia: interpretative phenomenological analysis. *Activities, Adaptation & Aging*. 2017;41(1):47-71. doi:10.1080/01924788.2016.1272392.

34. Hansen A, Hauge S, Hellesø R, Bergland Å. Purchasers' deliberations on psychosocial needs within the process of allocating healthcare services for older home-dwelling persons with dementia: a qualitative study. *BMC Health Serv Res*. 2018;18(1):746. Published 2018 Oct 1. doi:10.1186/s12913-018-3550-7

35. Harkey J. Safety first for a homebound patient with dementia. *Home Healthc Nurse*. 2014;32(10):601-602. doi:10.1097/NHH.0000000000000157

36. Harris PB. Hearing and healing the hurts of dementia, part 1: the experience of living alone with early stage Alzheimer’s disease: what are the person’s concerns? *Alzheimer’s Care Quarterly*. 2006;7(2):84-94. Accessed December 5, 2024. https://research-ebsco-com.ezproxy1.lib.asu.edu/linkprocessor/plink?id=e9127721-c8a5-3ab4-8c65-4a72025a9ca9

37. Harwood DG, Barker WW, Ownby RL, Duara R. Clinical characteristics of community-dwelling black Alzheimer's disease patients. *J Natl Med Assoc*. 2000;92(9):424-429.

38. Henderson C, Knapp M, Martyr A, et al. The Use and Costs of Paid and Unpaid Care for People with Dementia: Longitudinal Findings from the IDEAL Cohort. *J Alzheimers Dis*. 2022;86(1):135-153. doi:10.3233/JAD-215117

39. Herron RV, Rosenberg MW. "Not there yet": Examining community support from the perspective of people with dementia and their partners in care. *Soc Sci Med*. 2017;173:81-87. doi:10.1016/j.socscimed.2016.11.041

40. Honjo Y, Kawasaki I, Nagai K, Harada S, Ogawa N. Living Arrangements and Education Duration Associated With Memory Clinic Attendance in Alzheimer's Disease. *J Appl Gerontol*. 2023;42(7):1397-1403. doi:10.1177/07334648231155442

41. Isik AT, Soysal P, Kaya D, Usarel C. Triple test, a diagnostic observation, can detect cognitive impairment in older adults. *Psychogeriatrics*. 2018;18(2):98-105. doi:10.1111/psyg.12289

42. Keady J. Living alone with dementia. *Br J Nurs*. 1994;3(13):648-650. doi:10.12968/bjon.1994.3.13.648

43. Kerpershoek L, de Vugt M, Wolfs C, et al. Is there equity in initial access to formal dementia care in Europe? The Andersen Model applied to the Actifcare cohort. *Int J Geriatr Psychiatry*. 2020;35(1):45-52. doi:10.1002/gps.5213

44. Khanassov V, Rojas-Rozo L, Sourial R, Yang XQ, Vedel I. Needs of patients with dementia and their caregivers in primary care: lessons learned from the Alzheimer plan of Quebec. *BMC Fam Pract*. 2021;22(1):186. Published 2021 Sep 15. doi:10.1186/s12875-021-01528-3

45. Kirk E, Burrows L, Kent B, Abbott R, Warren A. Facilitators and barriers to remaining at home for people with dementia who live alone: a protocol for a systematic review of qualitative evidence. *JBI Database System Rev Implement Rep*. 2016;14(4):20-29. doi:10.11124/JBISRIR-2016-2550

46. Kolanowski A, Fortinsky RH, Calkins M, et al. Advancing Research on Care Needs and Supportive Approaches for Persons With Dementia: Recommendations and Rationale. *J Am Med Dir Assoc*. 2018;19(12):1047-1053. doi:10.1016/j.jamda.2018.07.005

47. Lee YJ, Johnston DM, Reuland M, Lyketsos CG, Samus Q, Amjad H. Reasons for Hospitalization while Receiving Dementia Care Coordination through Maximizing Independence at Home. *J Am Med Dir Assoc*. 2022;23(9):1573-1578.e2. doi:10.1016/j.jamda.2021.12.044

48. Lin HR, Otsubo T, Imanaka Y. Survival analysis of increases in care needs associated with dementia and living alone among older long-term care service users in Japan. *BMC Geriatr*. 2017;17:182. doi:10.1186/s12877-017-0555-8.

49. Mahon A, Kirwan R, Mackey L, et al. 168 Exploring Physical Activity and Sleep in Community-dwelling People with Dementia and their Association with Cognitive Function and Quality of Life...67th Annual & Scientific Meeting of the Irish Gerontological Society, Innovation, Advances and Excellence in Ageing, 26–28 September 2019, Cork, Ireland. *Age & Ageing*. 2019;48:iii17-iii65. doi:10.1093/ageing/afz103.100

50. Malloy P, McLaughlin NCR. Everyday functioning in dementia and mild cognitive impairment. In: Marcotte TD, Grant I, eds. *Neuropsychology of everyday functioning*The Guilford Press; 2010:264-279, 477 Pages. http://login.ezproxy1.lib.asu.edu/login?url=https://www.proquest.com/books/everyday-functioning-dementia-mild-cognitive/docview/622109395/se-2

51. Mansfield E, Cameron E, Carey M, et al. Prevalence and Type of Unmet Needs Experienced by People Living with Dementia. *J Alzheimers Dis*. 2022;87(2):833-842. doi:10.3233/JAD-215183

52. Matsuoka T, Ismail Z, Imai A, et al. Relationship between loneliness and mild behavioral impairment: Validation of the Japanese version of the MBI checklist and a cross-sectional study. *J Alzheimer's Dis.*2024;97(4):1951-1960. doi:https://doi.org/10.3233/JAD-230923

53. Mazurek J, Szcześniak D, Lion KM, Dröes RM, Karczewski M, Rymaszewska J. Does the Meeting Centres Support Programme reduce unmet care needs of community-dwelling older people with dementia? A controlled, 6-month follow-up Polish study. *Clin Interv Aging*. 2019;14:113-122. Published 2019 Jan 11. doi:10.2147/CIA.S185683

54. Mazurek J, Szcześniak D, Urbańska K, Dröes RM, Rymaszewska J. Met and unmet care needs of older people with dementia living at home: Personal and informal carers' perspectives. *Dementia (London)*. 2019;18(6):1963-1975. doi:10.1177/1471301217733233

55. Mejia-Arango S, Garcia-Cifuentes E, Samper-Ternent R, Borda MG, Cano-Gutierrez CA. Socioeconomic Disparities and Gender Inequalities in Dementia: a Community-Dwelling Population Study from a Middle-Income Country. *J Cross Cult Gerontol*. 2021;36(1):105-118. doi:10.1007/s10823-020-09418-4

56. Michalowsky B, Rädke A, Scharf A, et al. Healthcare Needs Patterns and Pattern-Predicting Factors in Dementia: Results of the Comprehensive, Computerized Unmet Needs Assessment from the Randomized, Controlled Interventional Trial InDePendent. *J Alzheimers Dis*. 2024;100(1):345-356. doi:10.3233/JAD-240025

57. Michalowsky B, Xie F, Eichler T, et al. Cost-effectiveness of a collaborative dementia care management-Results of a cluster-randomized controlled trial. *Alzheimers Dement*. 2019;15(10):1296-1308. doi:10.1016/j.jalz.2019.05.008

58. Miranda-Castillo C, Woods B, Galboda K, Oomman S, Olojugba C, Orrell M. Unmet needs, quality of life and support networks of people with dementia living at home. *Health Qual Life Outcomes*. 2010;8:132. Published 2010 Nov 12. doi:10.1186/1477-7525-8-132

59. Miyamae F, Taga T, Okamura T, Awata S. Toward a society where people with dementia 'living alone' or 'being a minority group' can live well. *Psychogeriatrics*. 2022;22(4):586-587. doi:10.1111/psyg.12836

60. Mukaetova-Ladinska EB. Silent lives: why do we fail community-dwelling people with dementia?. *Age Ageing*. 2017;46(3):341-343. doi:10.1093/ageing/afx028

61. Nakanishi M, Igarashi A, Ueda K, et al. Costs and Resource Use Associated with Community-Dwelling Patients with Alzheimer's Disease in Japan: Baseline Results from the Prospective Observational GERAS-J Study. *J Alzheimers Dis*. 2020;74(1):127-138. doi:10.3233/JAD-190811

62. Newhouse BJ, Niebuhr L, Stroud T, Newhouse E. Living alone with dementia: innovative support programs. *Alzheimer's Care Quart*. 2001;2(2):53-61.

63. Nygård L, Starkhammar S. Telephone use among noninstitutionalized persons with dementia living alone: mapping out difficulties and response strategies. *Scand J Caring Sci*. 2003;17(3):239-249. doi:10.1046/j.1471-6712.2003.00177.x

64. Nygård L, Starkhammar S. The use of everyday technology by people with dementia living alone: mapping out the difficulties. *Aging Ment Health*. 2007;11(2):144-155. doi:10.1080/13607860600844168

65. O'Connor DW, Pollitt PA, Brook CP, Reiss BB, Roth M. Does early intervention reduce the number of elderly people with dementia admitted to institutions for long term care?. *BMJ*. 1991;302(6781):871-875. doi:10.1136/bmj.302.6781.871

66. O’ Shea E, O’ Shea E, Timmons S, Irving K. The perspectives of people with dementia on day and respite services: a qualitative interview study. *Ageing and Society*. 2020;40(10):2215-2237. doi:10.1017/S0144686X1900062X

67. Okamura T, Sugiyama M, Inagaki H, et al. Anticipatory anxiety about future dementia-related care needs: towards a dementia-friendly community. *Psychogeriatrics*. 2019;19(6):539-546. doi:10.1111/psyg.12433

68. Parker LJ, Fabius C. Who's Helping Whom? Examination of Care Arrangements for Racially and Ethnically Diverse People Living With Dementia in the Community. *J Appl Gerontol*. 2022;41(12):2589-2593. doi:10.1177/07334648221120247

69. Phinney A, Chaudhury H, O'Connor DL. Doing as much as I can do: the meaning of activity for people with dementia. *Aging Ment Health*. 2007;11(4):384-393. doi:10.1080/13607860601086470

70. Portacolone E, Cohen AB. Living Alone With Dementia: A Reality Check. *Am J Geriatr Psychiatry*. 2024;32(11):1322-1324. doi:10.1016/j.jagp.2024.07.006

71. Reckrey JM, Leff B, Kumar RG, Yee C, Garrido MM, Ornstein KA. Home, but Not Homebound: A Prospective Analysis of Persons Living With Dementia. *J Am Med Dir Assoc*. 2022;23(10):1648-1652.e1. doi:10.1016/j.jamda.2021.12.029

72. Reckrey JM, Morrison RS, Boerner K, et al. Living in the Community With Dementia: Who Receives Paid Care?. *J Am Geriatr Soc*. 2020;68(1):186-191. doi:10.1111/jgs.16215

73. Sampath P (Kodi), Forbes DA, Barton S, Blake C. A Systematic Review of the Effectiveness of Interventions for Persons Living with Dementia Based in the Home or Community. *Perspectives: The Journal of the Gerontological Nursing Association*. 2015;38(2):6-19. Accessed December 5, 2024. https://research-ebsco-com.ezproxy1.lib.asu.edu/linkprocessor/plink?id=136be253-377a-3544-9d22-50a0ad657e07

74. Scharf A, Kleinke F, Michalowsky B, et al. Sociodemographic and Clinical Characteristics of People Living with Dementia and Their Associations with Unmet Healthcare Needs: Insights from the Baseline Assessment of the InDePendent Study. *J Alzheimers Dis*. 2024;99(2):559-575. doi:10.3233/JAD-231173

75. Sethuram C, Helmer-Smith M, Hammond J, Liddy C. Effective dementia care: what matters most to people living with dementia and five key components for delivery in primary care. *University of Toronto Medical Journal*. 2022;99(2):11-12. Accessed December 5, 2024. https://research-ebsco-com.ezproxy1.lib.asu.edu/linkprocessor/plink?id=0f953e25-b69d-3441-90c1-3d6cc4812c78

76. Smith GE, Lunde AM, Hathaway JC, Vickers KS. Telehealth home monitoring of solitary persons with mild dementia. *Am J Alzheimers Dis Other Demen*. 2007;22(1):20-26. doi:10.1177/1533317506295888

77. Swanwick GR, Coen RF, Maguire CP, et al. The association between demographic factors, disease severity and the duration of symptoms at clinical presentation in elderly people with dementia. *Age Ageing*. 1999;28(3):295-299. doi:10.1093/ageing/28.3.295

78. Tam MT, Martin S, Jiang YF, Machado A, Robillard JM. "Dementia Doesn't Mean That Life Doesn't Have More Wonderful Things Ahead": A Qualitative Study Evaluating a Canadian Dementia Support Services Program. *Can Geriatr J*. 2024;27(2):116-125. Published 2024 Jun 3. doi:10.5770/cgj.27.698

79. Thoma-Lürken T, Bleijlevens MHC, Lexis MAS, de Witte LP, Hamers JPH. Facilitating aging in place: A qualitative study of practical problems preventing people with dementia from living at home. *Geriatr Nurs*. 2018;39(1):29-38. doi:10.1016/j.gerinurse.2017.05.003

80. Tierney L, Doherty K, Breen J, Courtney-Pratt H. Community expectations of a village for people living with dementia. *Health Soc Care Community*. 2022;30(6):e5875-e5884. doi:10.1111/hsc.14018

81. Ura C, Inagaki H, Sugiyama M, et al. A neighbour to consult with is important in dementia-friendly communities: associated factors of self-efficacy allowing older adults to continue living alone in community settings. *Psychogeriatrics*. 2024;24(2):518-520. doi:10.1111/psyg.13054

82. van der Roest HG, Meiland FJ, Comijs HC, et al. What do community-dwelling people with dementia need? A survey of those who are known to care and welfare services. *Int Psychogeriatr*. 2009;21(5):949-965. doi:10.1017/S1041610209990147

83. von Kutzleben M, Schmid W, Halek M, Holle B, Bartholomeyczik S. Community-dwelling persons with dementia: what do they need? What do they demand? What do they do? A systematic review on the subjective experiences of persons with dementia. *Aging Ment Health*. 2012;16(3):378-390. doi:10.1080/13607863.2011.614594

84. Weber SR, Pirraglia PA, Kunik ME. Use of services by community-dwelling patients with dementia: a systematic review. *Am J Alzheimers Dis Other Demen*. 2011;26(3):195-204. doi:10.1177/1533317510392564

85. Yeh TP, Chen HC, Ma WF. A Qualitative Exploration of the Needs of Community-Dwelling Patients Living with Moderate Dementia. *Int J Environ Res Public Health*. 2021;18(17):8901. Published 2021 Aug 24. doi:10.3390/ijerph18178901
